# Bucket-Handle Meniscal Tears Might Not Be an Urgency: The Time to Meniscus Repair Does Not Seem to Affect the Mid-Term Outcome—A Retrospective Study of Sixty Tears with a Mean Follow-Up of 6 Years

**DOI:** 10.3390/jcm13113048

**Published:** 2024-05-22

**Authors:** Philipp Schippers, Victoria Buschmann, Felix Wunderlich, Yama Afghanyar, Sebastian Fischer, Erik Wegner, Philipp Drees, Erol Gercek, Lukas Eckhard

**Affiliations:** 1 Department of Orthopedics and Traumatology, University Medical Center, Johannes Gutenberg University Mainz, 55131 Mainz, Germany; victoria.buschmann@unimedizin-mainz.de (V.B.); felix.wunderlich@unimedizin-mainz.de (F.W.); yama.afghanyar@unimedizin-mainz.de (Y.A.); erik.wegner@unimedizin-mainz.de (E.W.); philipp.drees@unimedizin-mainz.de (P.D.); erol.gercek@unimedizin-mainz.de (E.G.); eckhard@gelenke-kh.de (L.E.); 2 Department of Foot and Ankle Surgery, Berufsgenossenschaftliche Unfallklinik Frankfurt Am Main, 60389 Frankfurt, Germany; 3 Praxis für Gelenkchirurgie Bad Kreuznach, 55543 Bad Kreuznach, Germany

**Keywords:** bucket-handle meniscal tears, meniscus, knee arthroscopy

## Abstract

**Background**: Bucket-handle meniscal tears are mostly treated arthroscopically. However, there is no clear evidence as to whether the time to surgery impacts the outcome and whether or not lesions should be treated urgently. **Methods**: Sixty patients were interviewed about the current status of their knee with a mean follow-up of 6.1 years (SD = 3.5). Forty-one patients underwent meniscus repair, and fifteen patients received partial resections. The primary outcome was the rate of reoperation after meniscus repair. Secondary outcomes were pain at rest and during exercise, return to sports, and Tegner and Lysholm scores. **Results**: The average time to surgery was 14.4 days, with no significant impact of surgical timing on the rate of reoperation. Furthermore, no significant differences were found in pain levels, return to sports, or Tegner and Lysholm scores based on the timing of surgery. **Conclusions**: In our cohort, the time to surgery was not a prognostic factor for the reoperation rate or postoperative outcome in repairing bucket-handle meniscal tears. Therefore, arthroscopic repair should not be performed in an emergency setting but conducted after careful planning by experienced arthroscopy surgeons. Regarding the return to sports, postoperative factors such as rehabilitation protocols or surgical techniques could be more important than the time to surgery.

## 1. Introduction

Bucket-handle meniscal tears (BHMTs) are longitudinal, full-thickness tears in which the attachment at the anterior and posterior horn is still intact [1]. BHMTs can be considered the most severe form of meniscus tear [2]. The prevalence of BHMTs ranges from 9 to 26% of all meniscus tears [3,4]. They mainly occur in young athletes after a sudden twist, pivot, or turn of the knee [5]; thus, BHMTs are often associated with ligamentous injuries, predominantly of the anterior cruciate ligament (ACL) [6,7,8]. However, BHMTs can also occur in a degenerative setting with a progressive onset of symptoms [9,10].

The treatment of a BHMT is predominantly arthroscopic. If the knee is still locked, the reduction can be performed carefully under visualization. There are different techniques for the repair: all-inside sutures, inside-out sutures, outside-in sutures, and various combinations [11,12,13]. In cases where repair is not feasible or promising, a partial meniscectomy is performed. The pooled reoperation rate after the surgical treatment of a BHMT varies between 14.8 and 29.3% according to two systematic reviews [7,8] but was as high as 75% in one study [14]. While some studies suggest that failures occur primarily within two years [7], others suggest a more extended period [15]. Many factors might influence the outcome and failure rate, yet there seems to be no influence of patient age, sex, or repair technique [15,16]. 

Another significant factor that may impact the outcome is the duration between trauma and the surgical intervention. The German Guidelines for Meniscus Injuries (S2k-Leitlinie 033/006—Meniskuserkrankungen) recommend surgery for locked bucket-handle meniscal tears (BHMTs), but they do not specify how soon the surgery should be carried out [17]. Similarly, the 2019 ESSKA Meniscus Consensus Statement advises that surgery for BHMTs should be conducted “as early as possible”, yet it stops short of providing a tangible timeframe [18]. While some articles state that delayed surgery is an adverse prognostic factor for meniscus repair in general [14,19,20,21], others postulate that the time to surgery is not a major factor for healing [22,23]. 

The return to sports, not only for athletes, is a primary concern after meniscal injuries. It is thus essential to identify any factors that influence recovery and return to sports. 

In this study, we set out to analyze BHMTs treated at our department with a particular emphasis on the time to surgery. Our primary research question was as follows: is there a correlation between the time to surgery and the rate of mid-term meniscus survival? The secondary outcomes analyzed were pain at rest and during exercise, return to sports, and Tegner and Lysholm scores. We hypothesized that the time to surgery would play a role in the rate of reoperation and outcome. 

## 2. Materials and Methods

### 2.1. Cohort

**Figure 1:**
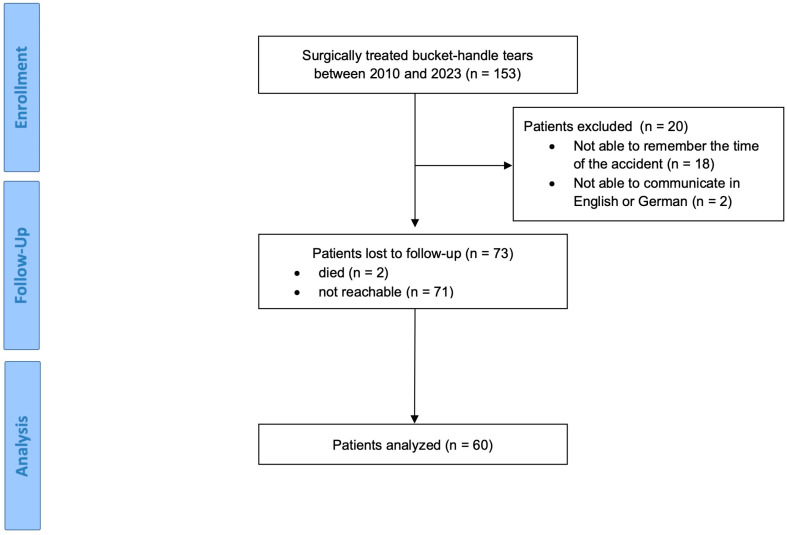
Flow chart (CONSORT).

After receiving institute board approval (N 2023-17118, Ethics Committee of Rhineland-Palatinate, Germany), a retrospective survey of our database for surgically treated bucket-handle tears of the medial or lateral meniscus was performed. Patients (n = 153) treated between 2010 and 2023 were enrolled. A total of 20 patients had to be excluded, and 73 were lost to follow-up. Thus, 60 patients (39%) were analyzed in this study (Figure 1). The mean follow-up was 6.1 years (SD = 3.5) and classified as mid-term, according to Ahmad et al. [24].

### 2.2. Surgical Technique

All patients were treated arthroscopically by attending orthopedic surgeons. Decision making, especially regarding whether or not resection or refixation should be performed, was carried out by the treating surgeon. Refixations were performed using all-inside sutures, inside-out sutures, outside-in sutures, or a combination. The postoperative rehab protocol for meniscal repair included a motion brace and partial weightbearing therapy of 10–20 kg for 6 weeks. For meniscal resection, the rehab protocol consisted of immediate, pain-adapted full weightbearing therapy without bracing. 

### 2.3. Outcome Evaluation

The participating patients were contacted via phone and interviewed about the status of their knee by one orthopedic surgeon. Patients were asked to confirm the date of the injury and surgery to calculate the delay of the surgical care, e.g., the time to surgery. Furthermore, the patients were asked whether they had to undergo the reoperation of the affected knee and for what reason (primary outcome). Furthermore, the patients reported current pain levels at rest and during exercise (VAS, Visual Analogue Scale of Pain) and whether they could return to sports (Yes or No). Finally, the present and pre-injury Tegner scores [25] and the current Lysholm score [26] were evaluated via phone. Further data, such as age and BMI at operation, were extracted from the hospital’s documentation.

### 2.4. Statistical Evaluation

For statistical analysis, GraphPad Prism v10.1.1 (GraphPad Software Inc., Boston, MA, USA) was used. The Shapiro–Wilk normality test was used to evaluate the distribution. For correlation analyses, Pearson’s correlation was used for normal distribution and Spearman’s correlation for non-Gaussian distribution. The Mann–Whitney test was used to compare two populations. *p*-values below 5% (*p* < 0.05) were considered significant.

## 3. Results

In this study, a total of sixty bucket-handle tears of the medial and lateral meniscus were retrospectively analyzed. The mean time to surgery was 14.4 days (range = 0–80; SD = 20.2). A total of 17 injuries concerned the lateral meniscus, 41 concerned the medial meniscus, and 2 were combined lesions of the lateral and medial menisci. In 19 patients, the BHTM was associated with an acute or chronic lesion of the ACL. Forty-five patients received a refixation (repair), and fifteen received a partial resection of the injured meniscus (Table 1). 

Among the fifteen partial resections, one patient received a further partial resection of the medial meniscus 8 months after the initial surgery. The other fourteen patients did not undergo a revision. 

**Figure 2:**
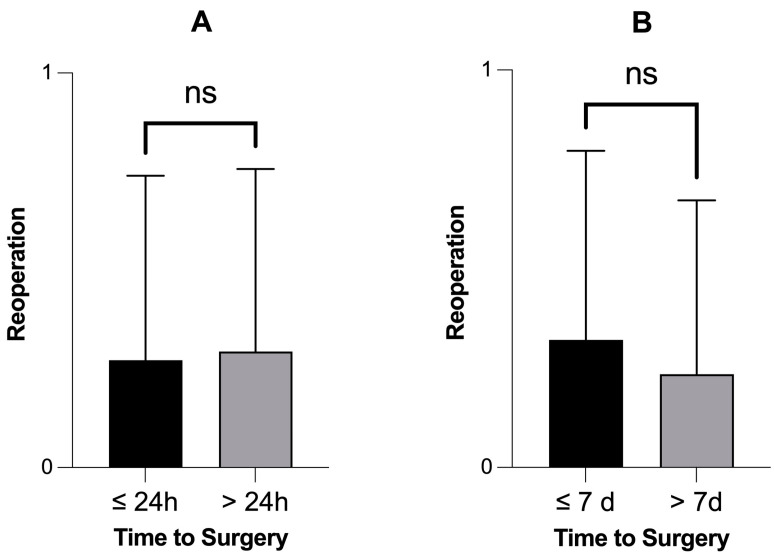
There was no difference in the rate of reoperation after meniscus repair when patients received an operation within 24 h (n = 13) or seven days (n = 34). The reoperation rate was not significantly different when patients received an operation within 24 h (**A**) or seven days (**B**). *p*-values > 0.05, Mann–Whitney test. ns = not significant.

Among the forty-five repairs, thirteen patients (28.8%) had to undergo a reoperation due to the initially injured meniscus after a mean duration of 2.3 years (SD = 2.1). The indication for revision was the failure of the repair. Eleven revisions concerned the medial and two concerned the lateral meniscus. Ten patients received a partial meniscus resection, and two underwent a refixation of the meniscus. Additionally, one patient underwent knee replacement. Among the reoperated patients, four had chronic ACL ruptures and one had an acute ACL rupture at the time of the initial meniscus tear. Patients who underwent reoperation had an average time to surgery of 10 days (SD = 15.7), while patients who did not undergo reoperation had an average time to surgery of 11.9 days (SD = 18.9). The difference in surgical delay was not statistically significant (*p* = 0.84, Mann–Whitney test). There was no difference in the rate of reoperations after meniscus repair (primary outcome) when patients were treated within 24 h or seven days (Figure 2). Using Spearman’s coefficient, there was also no correlation between the surgical delay and the reoperation rate: r = −0.03 (*p*-value = 0.83, confidence interval = −0.33–0.27).

Apart from the thirteen patients who underwent reoperation after meniscus repair, three patients were also operated on again, but this was not due to a failure of the initially operated meniscus: One patient received an ACL repair, and one patient underwent a reconstruction of the collateral ligaments; both operations were planned and staged procedures. Finally, one patient underwent an arthroscopic removal of a loose body. 

**Figure 3:**
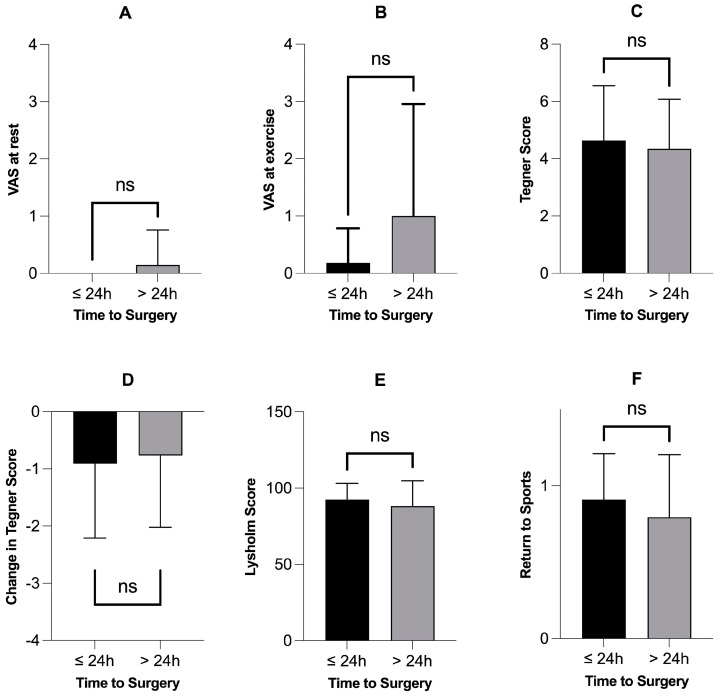
Patients receiving meniscus repair within 24 h (n = 11) did not have better outcome variables or scores. Subfigures (**A**–**F**) show different outcome variables and scores comparing the patients who were operated on within 24 h (n = 11) with those who were operated on after 24 h (n = 34). None of the six outcome variables showed a statistically significant difference: *p*-values > 0.05, Mann–Whitney test. VAS = Visual Analogue Scale for Pain; ns = not significant; h = hours.

**Figure 4:**
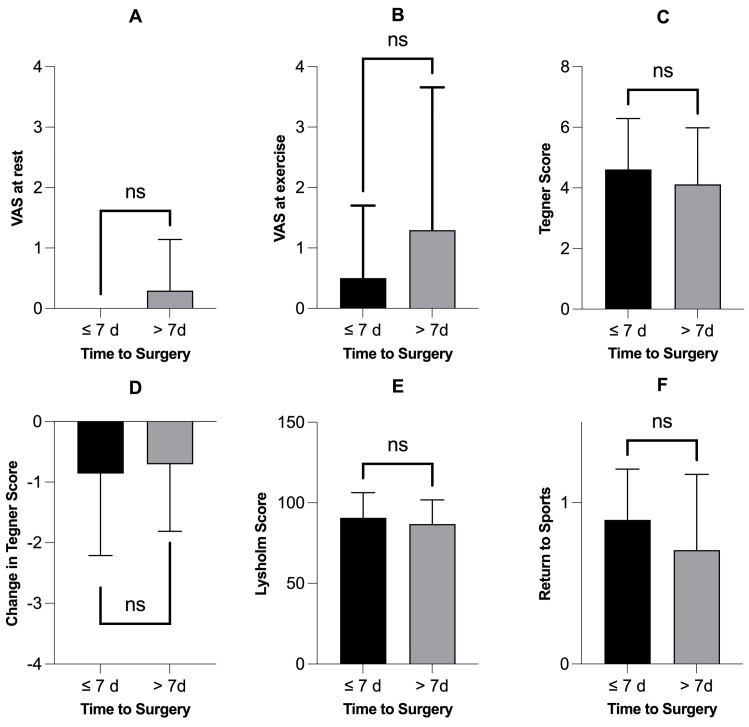
Patients receiving meniscus repair within 7 days (n = 28) did not have better outcome variables or scores. Subfigures (**A**–**F**) show different outcome variables and scores comparing the patients who were operated on within seven days (n = 28) with those who were operated on after one week (n = 17). None of the six outcome variables showed a statistically significant difference: *p*-values > 0.05, Mann–Whitney test. VAS = Visual Analogue Scale for Pain; ns = not significant; d = days.

There were no statistically significant differences in outcome variables among patients receiving meniscus repair when treated within 24 h (Figure 3) or seven days (Figure 4). Finally, there were no significant correlations between the time to surgery and different outcome variables and scores (Table 2).

## 4. Discussion

The most important finding of the present study is that no significant impact of the time to surgery on the outcome after the surgical repair of BHMTs was found. No difference was observed between patients treated within or after 24 h or within and after seven days. Likewise, there was no significant correlation between the time to surgery and pain (VAS), return to sports, or Tegner and Lysholm scores.

BHMTs, especially acute with the locking of the knee, often lead to emergency consulting. In theory, delayed surgery and the persistent locking of parts of the meniscus in the intercondylar notch could result in an extension of the lesion, fragmentation of the interposed tissue, ischemia, and, consequently, a reduced healing rate. Thus, time-critical diagnosis is desirable and has led to the development of CT protocols for the detection of BHMTs in case MRI is not available [27]. Older studies especially have highlighted the importance of the rapid treatment of all kinds of meniscus tears [19,20]. Athletes might be very concerned and demand immediate treatment, even on weekends, since they are highly dependent on a quick return to sports. Thus, it is important to provide athletes with accurate guidance; specifically, athletes should be informed that the time to surgery might be a less important factor and that rehabilitation protocols or surgical techniques could be more influential. 

Many studies have analyzed the outcome and failure rate of arthroscopically treated BHMTs [7]. Several investigations also mentioned the time from injury to surgery, with a mean surgical delay varying from 0.3 to 28 months [2,9,28,29,30,31,32,33,34,35,36,37,38]. However, several studies focused predominantly on surgical techniques [28,33,35,36,37,38]. Lucas et al. found no correlation between the time to surgery and outcome in a pediatric population [34]. Kaminski et al. and Saltzman et al. found no impact of the time to surgery on outcome in an adult population [9,32]. Cetinkaya et al. reported positive results even in neglected BHMTs with a mean surgical delay of 28 months [29]. According to Feng et al., there is no difference in outcome between patients treated within 12 months or after [30]. In contrast, Hupperich et al. reported significantly better outcomes for patients operated within seven days or later. In their study, further influential factors such as patient sex, preoperative activity level, and the number of sutures were identified. A total of 38 patients with a mean surgical delay of 1.5 months were analyzed [31]. In our population, the only notable difference when comparing patients operated on within seven days or after was the rate of return to sports. 

The authors’ preferred treatment in the case of a sudden locked knee consists of timely MRI imaging. In the case of a BHMT, the knee is immobilized, and planned surgery is performed during regular weekdays with a maximum presence of staff and well-trained arthroscopic surgeons. In our opinion, the benefits of the surgery performed by experienced surgeons outweigh immediate arthroscopic reduction and refixation by less experienced surgeons. 

This study has several limitations: First, there is potentially a blend of acute vs. chronic BHMTs, which might constitute different entities. Second, due to the long enrollment period, patients were treated by several different surgeons utilizing varying techniques. With only a limited number of patients available for some of these techniques, sub-analyses comparing technical aspects were not possible. Additionally, in the absence of clear guidelines, surgeon bias might have played an important role when deciding between resection or refixation, eventually impacting the healing rate. Since protocols and surgical experience might differ among clinical institutions, the results might not necessarily be generalizable. Furthermore, this study’s retrospective design, including the backward-looking collection of dates and scores, could represent another potential source of error. There was, for instance, no clear and consistent dislocation status of the meniscus available. Moreover, the follow-up was only conducted via phone; hence, the results are lacking a physical examination. Lastly, there is a potential bias of the analyzed patients due to a relatively high number of patients who were lost to follow-up. This can be partly explained by the long follow-up which goes up to 14 years in a young and dynamic population that is known for changing addresses. However, the absolute number of patients analyzed and the mean follow-up are higher than those in most studies available [7].

## 5. Conclusions

In our cohort of sixty patients, the time to surgery was not a prognostic factor for the reoperation rate or postoperative outcome in repairing bucket-handle meniscal tears. Therefore, arthroscopic repair should not be performed in an emergency setting but conducted after careful planning by experienced arthroscopy surgeons. Regarding the return to sports, postoperative factors such as rehabilitation protocols or surgical techniques could be more important than the time to surgery. Finally, it has to be noted that the variability in surgical techniques and the surgeon’s experience over the long enrollment period could introduce biases that might have affected the outcomes.

## Figures and Tables

**Table 1:** Patient demographics according to treatment.

	Refixation (n = 45)	Partial Resection (n = 15)	*p*-Value
Time to surgery	11.6 days (SD = 18.7)	22.9 days (SD = 22.8)	ns
Sex	17 women, 28 men	4 women, 11 men	
Age	32.5 years (SD = 11.2)	37.9 years (SD = 11.9)	ns
BMI	26.2 kg/m^2^ (SD = 6.7)	28.9 kg/m^2^ (SD = 8.8)	ns

Surgical delay (time between injury and operation) and patient demographics (sex, age, and BMI) are shown according to the treatment (refixation vs. partial resection) patients received. Both groups showed no statistical difference. A Mann–Whitney test was used to calculate *p*-values. ns = not significant (*p* > 0.05).

**Table 2:** Time to surgery does not correlate with outcome variables and scores.

	All Patients (n = 60)	Meniscus Repair (n = 45)
Time to Surgery vs.	Spearman’s Correlation Coefficient (r) Confidence Interval (CI); *p*-Value	
VAS at rest	r = 0.18 CI: −0.08–0.43; *p*-value: 0.16	r = 0.22 CI: −0.09–0.49; *p*-value: 0.15
VAS at exercise	r = 0.16 CI: −0.11–0.41; *p*-value: 0.23	r = 0.27 CI: −0.03–0.53; *p*-value: 0.07
Tegner Score	r = −0.11 CI: −0.37–0.15; *p*-value: 0.39	r = −0.17 CI: −0.45–0.14; *p*-value: 0.27
Change in Tegner Score	r = 0.07 CI: −0.20–0.32; *p*-value: 0.60	r = 0.00 CI: −0.30–0.30; *p*-value: 0.99
Lysholm Score	r = −0.19 CI: −0.43–0.08; *p*-value: 0.15	r = −0.18 CI: −0.46–0.13; *p*-value: 0.23
Return to Sport	r = −0.22 CI: −0.45–0.05; *p*-value: 0.10	r = −0.16 CI: −0.44–0.15; *p*-value: 0.30

Spearman’s correlation coefficient (r) was calculated between the surgical delay (time between trauma and surgery) and different outcome variables (VAS at rest, VAS at exercise, Tegner score, change in Tegner, Lysholm score, and return to sport). The change in Tegner score was calculated as the difference between the pre-injury and current scores.

## Data Availability

The raw data supporting the conclusions of this article are available from the authors upon request.
